# Using Artificial Intelligence Methods to Evaluate the Effect of the National Cytomegalovirus Awareness Month on the Content and Sentiment of Social Media Posts: Infodemiology Study

**DOI:** 10.2196/80922

**Published:** 2026-01-22

**Authors:** Tracy R Rosebrock, Zhen Yang, Lauren D'Arco, Tapan Pathak, Rebecca Vislay-Wade, Karen Fowler, John Diaz-Decaro, Colin Kunzweiler

**Affiliations:** 1 Department of Health Science, Department of Biology School of Arts and Sciences Stonehill College North Easton, MA United States; 2 Moderna Therapeutics (United States) Cambridge, MA United States; 3 Department of Pediatrics Heersink School of Medicine University of Alabama at Birmingham Birmingham, AL United States

**Keywords:** cytomegalovirus, social media, public health, health communication, sentiment analysis, artificial intelligence

## Abstract

**Background:**

The month of June has been recognized as the National Cytomegalovirus (CMV) Awareness Month since 2011 in the United States. Established by government resolution, the goal is to increase awareness and reduce the incidence of congenital CMV infection, a leading cause of preventable birth defects and developmental disabilities. Social media is a powerful tool to support public health by making health information easily accessible. With an estimated 246 million users in the United States and more than half of adults seeking health information through such platforms, social media offers an unparalleled opportunity to promote CMV awareness and prevention.

**Objective:**

This study aimed to evaluate social media messaging before, during, and after the National CMV Awareness Month to assess how the campaign influenced messaging patterns and sentiment related to specific CMV health topics.

**Methods:**

Publicly available posts on Twitter/X from May to August 2023 that contained at least one of the five most used CMV-related hashtags were collected using a media monitoring platform. The dataset was preprocessed using a customized Bidirectional Encoder Representations from Transformers tokenizer and a language detection package to remove irrelevant and non-English posts. Validated and artificial intelligence (AI) methods (Cohen κ=0.69) were used to determine the thematic content of posts (N=14,900), such as awareness and prevention messaging, and to characterize the sentiment. Changes in post characteristics were measured in relation to the National CMV Awareness Month.

**Results:**

CMV-relevant post volume increased by 55% during the campaign month and returned to precampaign levels in July. Overall, academic/university researchers were the most frequent authors, pediatrics was the most frequent population discussed, and vaccines were the most frequently mentioned prevention. Significant associations were observed between the month of post publication and the target audience (*χ*^2^_2_=144.3, *P*<.001), awareness or prevention messaging (*χ*^2^_2_=107.8, *P*<.001), and post sentiment (*χ*^2^_4_=163.6, *P*<.001). The intended audience of posts shifted toward the general population from scientists/health care professionals during the campaign month (adjusted Pearson residuals, *P*=.009). Awareness messaging increased in June 2023, particularly in relation to CMV transmission and disease burden, while prevention messaging decreased (adjusted Pearson residuals, *P*=.008). Finally, although posts were generally neutral in sentiment, a significant shift occurred toward a positive sentiment during the campaign month (adjusted Pearson residuals, *P*=.006), a sentiment that was more likely to engage the user (Kruskal-Wallis; *χ*^2^_2_=194.31, *P*<.001).

**Conclusions:**

The National CMV Awareness Month in 2023 shifted the digital CMV conversation toward public-facing messaging and raised awareness efforts. Although posts related to CMV prevention generally conveyed a positive sentiment, prevention messaging declined during the campaign. These findings highlight opportunities for future CMV social media initiatives to balance awareness with prevention through evaluation and strategic design using AI models to strengthen CMV public health communication and engagement.

## Introduction

### Background

Human cytomegalovirus (CMV) is a ubiquitous beta-herpesvirus with an estimated seroprevalence of 83% worldwide and 63% in the United States and a disease burden that disproportionally impacts disadvantaged and minoritized communities [1-4]. The virus is transmitted through direct contact with infectious body fluids, through organ/stem cell transplants, and transplacentally, which can result in congenial CMV infection [5,6]. Infection with CMV is lifelong and is typically subclinical in healthy individuals, though CMV may silently contribute to chronic conditions, such as cardiovascular disease [7], cognitive decline [8], neurologic disorders [9,10], anxiety and depression [11], immunosenescence [12], Guillain-Barré syndrome [13], certain malignancies (eg, glioblastoma multiforme) [14], and all-cause mortality [15]. In those who are immunocompromised, CMV infection can lead to acute disease and death [16,17].

When CMV is transmitted from mother to fetus during pregnancy, it is referred to as congenital cytomegalovirus (cCMV). In the United States, 1 in 200 infants is born with cCMV [18]. The majority of infants born with cCMV are asymptomatic without recognizable signs or symptoms at birth, while a subset (~10%) may be impacted by a wide range of signs and symptoms at birth [19]. Regardless of the presence or absence of symptoms at birth, all infants born with cCMV are at risk for developing long-term sequelae, such as sensorineural hearing loss, vision impairment, cerebral palsy, and developmental delays [20]. CMV is also a recognized cause of stillbirth and intrauterine fetal demise [21-24]. Despite the significant burden of cCMV [25,26], just 13% of women are aware of CMV, cCMV, or simple hygiene prevention practices that can reduce risk [27]. Given the limited effectiveness of pharmaceutical interventions and the lack of a licensed vaccine to prevent infection, public health education is of critical importance.

In 2011, the US Senate issued a resolution declaring June to be the National CMV Awareness Month, with explicit objectives to raise awareness of the dangers of CMV and to reduce the incidence of cCMV infections through education [28]. Today, eHealth, or the delivery of health information digitally, is recognized as an important frontier for public health education [29]. Social media is an important tool for public health education in the United States, where ~246 million people, or 73% of the population, are active users [30]. Among US adults, 55% report using social media at least occasionally to seek health information, and for the ~95 million US users of Twitter/X, the average daily use is greater than 30 minutes per day on the platform [31,32]. These platforms offer a valuable opportunity to disseminate accurate information and raise awareness about cCMV and prevention.

The National CMV Awareness Month is primarily promoted by the National Cytomegalovirus Foundation (NCMVF), advocacy groups of families affected by cCMV, and state and national public health agencies [33,34]. Shared goals are to increase public awareness and educate on prevention behaviors, while advocacy groups also promote expanded CMV screening and research. The awareness campaign is visible on social media through educational graphics, family stories, and hashtags, such as #stopCMV and #CMVawareness, to spread information and engage the public. Although CMV-specific campaign activity is described by advocacy organizations, prior public health infodemiological research has not systematically evaluated how these campaigns function on social media platforms.

Natural language processing (NLP) and sentiment analysis are novel, powerful, and essential tools in public health for monitoring public opinions toward health-related topics and identifying potential areas and concerns. For example, recent research has used Bidirectional Encoder Representations from Transformers (BERT) to analyze social media posts to understand public opinions toward the impact of the COVID-19 pandemic on social life [35] and sentiments expressed during an outbreak in Uganda [36]. Despite coordinated social media campaigns dedicated to CMV awareness, to date, sentiment analysis has not been conducted regarding this disease.

Beyond BERT-based approaches, recent infodemiology studies have increasingly incorporated large language models (LLMs) to analyze high-volume Twitter/X data for public health insight. For example, artificial intelligence (AI) models have been used to classify tweets related to conjunctivitis outbreaks and estimate epidemic signals, and LLM-driven sentiment and substance-use detection models have been applied to opioid-related social media data [37,38]. Such studies demonstrate the expanding role of generative and transformer-based models in characterizing eHealth discourse, providing methodological precedent for applying LLMs to evaluate public health messaging on Twitter/X.

Although survey-based studies have assessed CMV awareness and attitudes toward prenatal and neonate CMV screening [39,40], formal infodemiology or eHealth investigations, particularly those evaluating social media messaging related to CMV awareness and prevention, have not been conducted. This study represents the first systematic evaluation of CMV-related discourse on social media, characterizing Twitter/X messaging during the National CMV Awareness Month and assessing how this communication aligns with goals set forth by the US Senate and public health stakeholders. Although prior infodemiology research has analyzed other public health campaigns or disease-related discourse using transformer models and LLMs, no studies have examined CMV or cCMV infections, and none have assessed a nationally recognized awareness-month campaign. By leveraging an LLM to classify, summarize, and evaluate campaign-related tweets, this study used emerging LLM-based methods to fill an important gap in the literature on digital CMV education and awareness.

### Study Objective

The aim of this study was to evaluate the messaging of CMV-related posts on Twitter/X before, during, and after the National CMV Awareness Month in June 2023 (ie, May-August 2023).

## Methods

### Data Aggregation

Investigators collected social media posts from the Twitter/X platform (Twitter became X on July 23, 2023) for May-August 2023, representing the months immediately preceding and following the National CMV Awareness Month in June using Keyhole, a subscription-based, commercial social listening and analytics platform. Keyhole provides real-time tracking, historical backfill, and reach/impression estimates based on post volume and author follower counts.

To be eligible for this initial download, social media posts must have included one or more of the following five hashtags: #stopCMV, #cCMV, #CMV, #CMVawareness, and #cytomegalovirus. No additional filters were applied. These hashtags were identified by two independent reviewers, who manually assessed Twitter/X posts referencing “cytomegalovirus” or “CMV” using the site’s search function. The most recent posts were assessed weekly from March 1 to April 30, 2023. From relevant posts that included hashtags, the five hashtags used in this study were the most frequently used and captured all posts in the assessment window that used hashtags.

In addition to the social media post text, user information (eg, username, biography, number of followers, and location) and dissemination-related metrics (eg, number of reposts, number of likes, and number of comments) were downloaded for each post. More than 30,000 social media posts were downloaded based on the aforementioned criteria.

### Data Preparation

Non-English language posts were detected using the language-detecting Python package, *langdetect*, and removed during preliminary data-cleaning procedures. Among the more than 20,000 English-language social media posts that remained, posts were first cleaned to remove noise and other nuisance text (eg, URLs, emojis, and hashtag [#] and mention [@] symbols). A fine-tuned text classifier was next applied to identify relevant social media posts and exclude irrelevant social media posts from analyses [41]. Briefly, a customized BERT tokenizer was used to complete word-level tokenization of all social media posts. Reading social media posts from both left and right, the BERT tokenizer can understand the language context and flow of words based on a given word’s surroundings. By default, BERT tokenizes, or breaks down, words into subword units. For example, the term “congenital” may be segmented into the subword units “con,” “##gen,” and “##ital” (the “##” prefix indicates that the subword is a continuation of the previous token unit). However, during exploratory data analysis, it was noticed that certain keywords, and in particular abbreviations, were segmented into individual characters (eg, “CMV” was segmented into “C,” “##M,” and “##V). To ensure meaningful pretraining, the BERT tokenizer was customized by manually adding a dictionary of domain-specific words (eg, “congenital,” “cCMV,” and “CMV”) that would be identified as complete words, as opposed to subword units. We compared model performance using the default bert-base-cased tokenizer versus the customized tokenizer, each fine-tuned on the same manually labeled dataset. Both models achieved comparable accuracy (95%), indicating that the custom dictionary did not materially affect overall performance. Additional methodological details are provided in Multimedia Appendix 1.

### Descriptive Analyses

Numerous descriptive analyses were conducted. The date of social media posts was analyzed to describe the number of posts per month for the May-August 2023 period. The location of each relevant, English-language social media post (when such information was available) was summarized at the country level (data not shown), as well as at the state level for posts originating in the United States. User categories (eg, university/academic researchers, news/journal/public health/education, and physicians/health care/hospitals) were summarized for the top 20 users with the most social media posts or most followers during the study period. The biography data supplied in the author’s Twitter/X biosketch were used to determine their user category, or if a biography statement was not provided, the author was located using a web search and categorized using available data. Metrics of impact, including the number of followers, reposts, and “likes,” were also summarized.

### Theme (Aspect) Classification

Following the identification of relevant social media posts, investigators developed a master prompt for Moderna’s internal ChatGPT AI tool (mChat), as shown in Multimedia Appendix 2, to annotate the specific aspects contained within each post. mChat serves as a pass-through to the OpenAI application programming interface (API), does not modify model behavior, and directly calls the OpenAI API using company credentials.

Aspects were first prespecified by two independent investigators and were grouped according to four broad categories: population discussed (the subject of the social media post; eg, “women of reproductive age,” “parents,” and “pediatric patients”); awareness and knowledge (a list of clinical and nonclinical terms related to CMV; eg, “seroconversion,” “newborn screening,” “National CMV Foundation,” and “parental education”); prevention (a list of terms describing preventive measures related to CMV; eg, “hygiene measures,” “antiviral treatment,” and “vaccines”); and general CMV information (generic terms; eg, “safety,” “efficacy,” and “tolerability”). In addition, the perceived target audience (eg, general population, scientists/health care professionals) was annotated (see Table S1 in Multimedia Appendix 3 for a complete list of prespecified aspects within each thematic category). Investigators manually classified prespecified aspects for approximately 90 social media posts. The ChatGPT model then used the manually classified social media posts for few-shot learning for aspect classification. Although 90 manually classified posts represented a small subset of the >10,000 posts analyzed, the approach ensured complete coverage of all prespecified aspects with representation in at least 2 example posts, making the number of manually classified posts commensurate with the list of aspects of interest (Table S1 in Multimedia Appendix 3).

To load the full dataset stored in a Microsoft Excel spreadsheet, the tweets were imported into data frames using the Python *pandas* library, and only the tweet-text column was used as input for annotation. The master prompt (Multimedia Appendix 2) contained (1) step-by-step instructions for identifying CMV-related aspects, segmenting text, and assigning sentiment, while maintaining aspect integrity, and (2) the set of approximately 90 manually annotated tweets used as few-shot examples. The model temperature was set to 0 to promote deterministic outputs. Responses were requested in JSON format (keys included aspects, aspect segment, sentiment toward each aspect, and overall sentiment; see sentiment methods in the *Sentiment and other Statistical Analyses* section). Each tweet was processed with up to five retries to ensure valid JSON formatting. No manual prompt revisions were made between batches, and no additional model fine-tuning was performed. Reproducibility was supported by fixing model parameters (temperature=0), using a single master prompt for all tweets, processing tweets independently, and allowing up to five retries for valid JSON output. To enhance transparency, the prompt instructed ChatGPT to include additional keys in the JSON output, identifying the specific text segments that contributed to each aspect or sentiment label. This allowed investigators to trace which words or phrases informed each annotation. Because annotations were produced via prompt-based generation rather than model training, no explicit class-balancing techniques were applied.

### Sentiment and Other Statistical Analyses

Social media posts and specific aspects identified within the posts were assigned a sentiment (ie, positive, neutral, or negative). Four independent, blinded reviewers assigned a sentiment to 97 randomly selected post texts outside of the dataset with moderate agreement, with an interrater reliability score of 0.56 (Fleiss κ; “moderate” defined as 0.41-0.6 [42]). The ChatGPT model was then provided with the scored posts for few-shot learning and asked to assign a sentiment to social media posts included in the analysis dataset. Following assignment, text from 50 posts scored by ChatGPT was evaluated for sentiment by the same four independent and blinded reviewers. Moderate interrater reliability was measured at 0.51 (Fleiss κ). The interrater reliability score between the sentiment assigned by most reviewers and ChatGPT was 0.69 (Cohen κ), indicating substantial agreement between reviewers and ChatGPT (“substantial” defined as 0.61-0.8 [42]). Of the 10 posts, or 20%, where human-AI agreement was not observed, 90% of posts were scored neutral by one party and either positive or negative by the other, indicating that minor discrepancies rather than large errors (positive versus negative) explain the discordance.

Statistical analyses (Fleiss κ, Cohen κ, chi-square tests with Bonferroni correction, Kruskal-Wallis with Bonferroni correction) were conducted using Excel or Datatab. Chi-square tests were applied only to independent categorical variables. Individual posts often contained multiple dependent variables (eg, multiple hashtags; multiple aspects within a category, such as target audience, awareness, or prevention messaging). These attributes were excluded from tests requiring variable independence. Adjusted Pearson residuals were calculated for chi-square cross-tabulations, and Bonferroni correction was applied to adjust *P* values for multiple comparisons to set the critical threshold for significance.

### Ethical Considerations

This study used only publicly available text from social media posts on Twitter/X. The research team did not interact with post authors or collect private information. All data were processed to remove or avoid inclusion of identifiable information and are reported only in aggregate. This study did not constitute human subjects research and therefore did not require review or approval by an institutional review board per Federal Regulations for the Protection of Human Research Subjects (45 CFR 46.104(d)(4)(i); [43]).

## Results

### Data Aggregation and Processing

**Figure 1:**
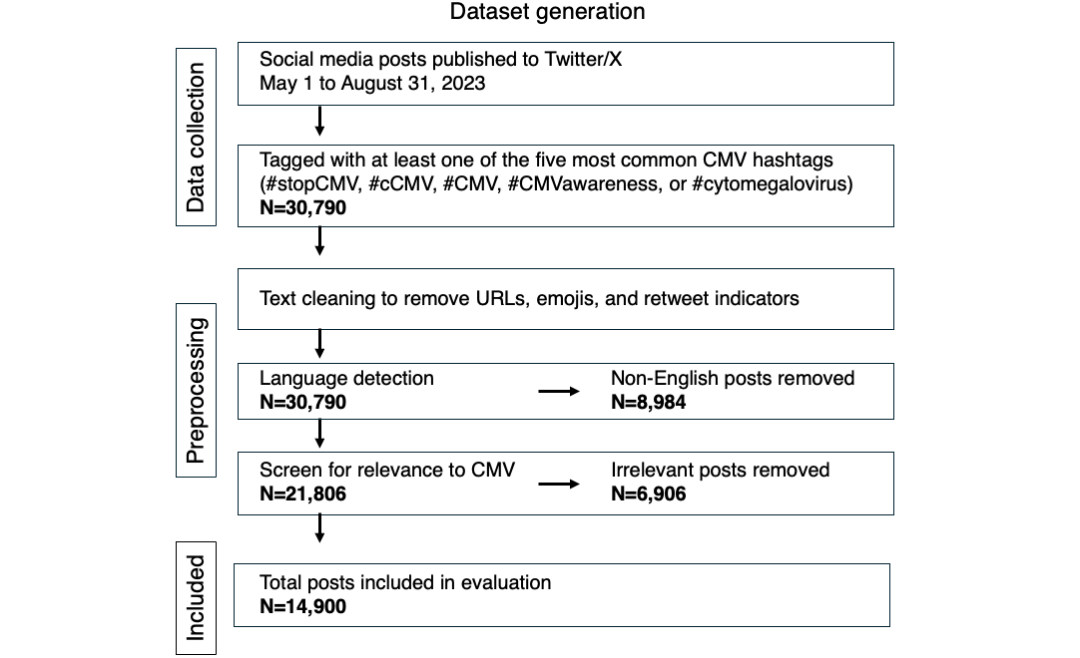
Flowchart of the dataset collection, preprocessing, and inclusion steps. cCMV: congenital cytomegalovirus; CMV: cytomegalovirus.

Between May 1 and August 31, 2023, a total of 30,790 public posts published to Twitter/X were tagged with one or more of the five most common hashtags used to reference CMV or CMV disease. Several hundred posts were cotagged with hashtags unrelated to CMV (eg, #SHREKINU, a cryptocurrency) or used a CMV-related hashtag to indicate a non-CMV topic (eg, #CMV=commercial motor vehicle). Additionally, nearly one-third of posts were written in a language other than English. To focus subsequent analyses, the 30,790 posts were preprocessed to (1) remove noise elements, such as URLs and emojis; (2) filter out non-English posts; and (3) extract posts relevant to CMV or CMV disease (Figure 1).

### Descriptive Analyses

**Figure 2:**
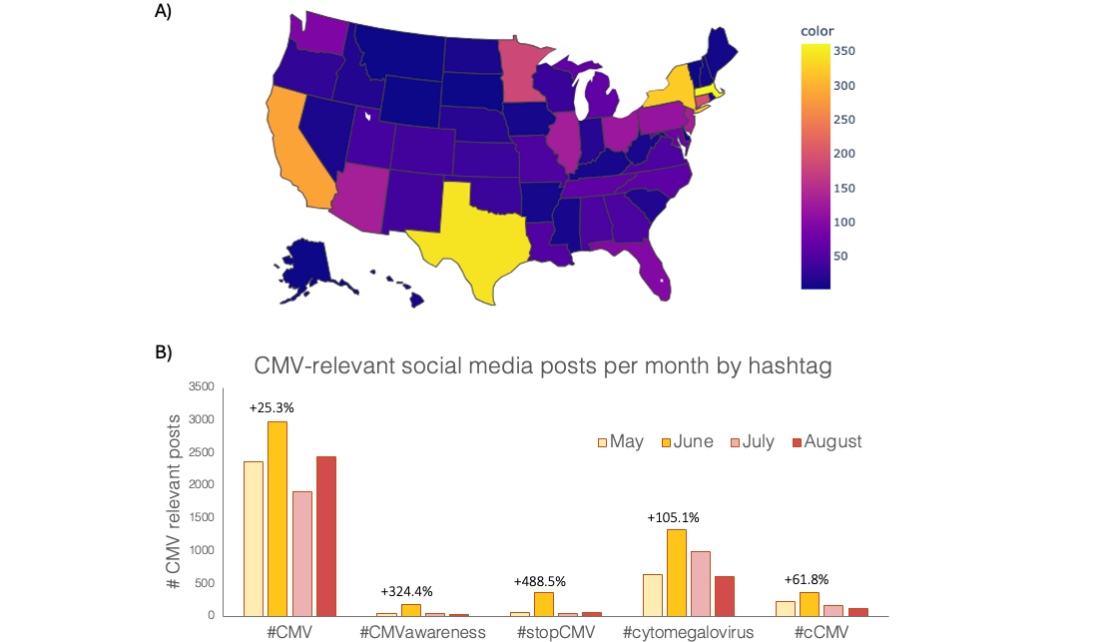
Descriptive statistics. Quantification of CMV-relevant public posts (A) published to Twitter/X at the US state level and (B) sorted by hashtag by month (May-August 2023). Noted percentages reference the change in post number from May to June (National CMV Awareness Month). cCMV: congenital cytomegalovirus; CMV: cytomegalovirus.

Overall, 14,900 posts were analyzed. There were 3336 (22.4%) social media posts in May 2023, which increased by 55% to 5180 (34.8%) posts in June 2023 (coinciding with the National CMV Awareness Month in the United States), and decreased during both July 2023 (n=3124, 21%) and August 2023 (n=3260, 21.9%), as shown in Table S2 in Multimedia Appendix 3. Social media posts most frequently originated from the United States (n=3858, 25.9%). The next most frequent countries included Canada (n=594, 4%), the United Kingdom (n=535, 3.6%), India (n=384, 2.6%), and Australia (n=370, 2.5%). In the United States, among posts with known geographic locations, Massachusetts, Texas, and New York had the highest number of posts (Figure 2A). A more detailed analysis of posts grouped by hashtag revealed additional trends. The hashtag #CMV was the most used (n=9674, 64.9%), followed by #cytomegalovirus (n=3558, 23.9%), #cCMV (n=859, 5.8%), #stopCMV (n=529, 3.6%), and #CMVawareness (n=280, 1.9%), as shown in Figure 2B. With respect to the National CMV Awareness Month, an increase in posts was observed from May to June for all five hashtags. The largest proportional increases occurred with #stopCMV (488.5%, from n=61, 0.4%, to n=359, 2.4%, posts) and #CMVawareness (+324.4%; from n=41, 0.3%, to n=174, 1.2%, posts), possibly driven by national advocacy organizations.

To understand who is publishing CMV-relevant content on social media, the authors of posts containing each CMV-related hashtag were analyzed. Because a single post may include multiple hashtags, authors could appear across several hashtag groups. For each hashtag, authors were ranked by the total number of posts they published, and the top 20 most frequent authors were identified. These authors were then categorized by author category based on their profile affiliation. To describe overall trends, the top 20 authors from each hashtag were combined into a single dataset, with duplicate users removed. The most common author category was “university/academic researchers,” followed by “news/journal/public health/education and “physicians/health care/hospitals” (Table S3 in Multimedia Appendix 3).

Hashtag-specific trends included the nearly equal representation of most CMV stakeholders under #CMV, in contrast to skewed author distributions for #CMVawareness and #cytomegalovirus. The top 20 authors scored by total followers were also assessed. Unsurprisingly, the most common follower category was “news/journal/public health/education” (Table S3 in Multimedia Appendix 3).

### Classification of Thematic (Aspects) Content

**Figure 3:**
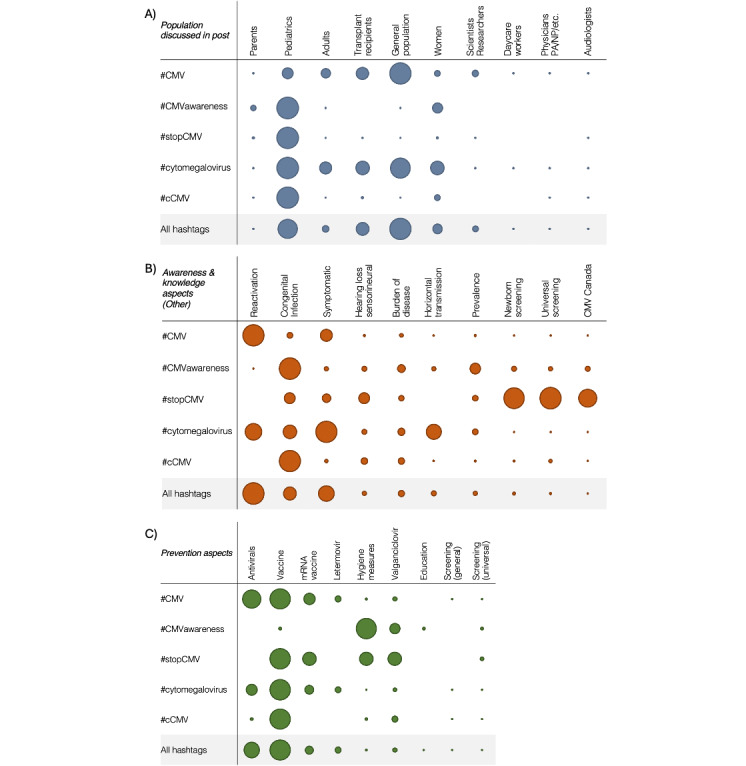
Relative comparison of the number of posts by aspect for each hashtag or aggregated. (A) Population discussed in a post, (B) awareness aspects other than CMV or cCMV, and (C) CMV prevention aspects. The largest circle in a row represents the highest number of posts with that aspect for that hashtag. All other circles are proportional to this reference. cCMV: congenital cytomegalovirus; CMV: cytomegalovirus; mRNA: messenger RNA.

Social media posts were also evaluated for thematic content. Five broad thematic categories (target audience, population discussed, awareness and knowledge, prevention, and general CMV information) were created and populated with specific aspects to be identified within the posts. Overall, the “scientific/health care professionals” category was the most frequent target audience of social media posts (n=7575, 50.8%); however, this varied by hashtag (Table S4 in Multimedia Appendix 3). The “general population” category was the most frequent population discussed across all five hashtags (n=2727, 18.3%), followed by “pediatrics” (n=2456, 16.5%), as shown in Figure 3A and in Table S5 in Multimedia Appendix 3. Transplant recipients (n=1636, 11%) were the third-most frequent population discussed, though they were nearly exclusively mentioned in #CMV and #cytomegalovirus posts. “Women” (n=1141, 7.7%), “adults” (n=784, 5.3%), and “scientists” (n=691, 4.6%) were also frequently discussed; all other prespecified aspects related to the “population discussed” category were identified in fewer than 2235 (15%) of the 14,900 social media posts evaluated.

Approximately 50 aspects were prespecified for the “awareness and knowledge” category (Table S1 in Multimedia Appendix 3). Perhaps expectedly, “CMV” was overwhelmingly the most frequently identified aspect (n=10,929, 73.3%, posts), with “cCMV” being the second-most frequently identified aspect (n=1598, 10.7%), as shown in Table S6 in Multimedia Appendix 3. The frequency of aspects other than “CMV” and “cCMV” varied by hashtag, with the third-most frequent aspect being “reactivation” for #CMV and #cytomegalovirus, “congenial infection” for #CMVawareness and #cCMV, and “newborn/universal screening” for #stopCMV (Table S7 in Multimedia Appendix 3 and Figure 3B). Although “prevention” aspects were included in just 2593 (17.4%) of the 14,900 posts, the most frequent preventions identified included “vaccines” (n=1115, 7.5%), “antiviral treatment” (n=849, 5.7%), “mRNA vaccine” (n=453, 3%), and “Letermovir” (n=332, 2.2%), as shown in Figure 3C and in Table S8 in Multimedia Appendix 3. “Hygiene measures” and the specific antiviral treatment “Valganciclovir” were less common overall (n=114, 0.8%, and n=214, 1.4%, respectively), though these were the most frequent “prevention” aspects within #CMVawareness posts. In the final category, which included terms reflecting “general CMV information,” “immune response” was the most frequently identified aspect (n=533, 3.6%), which was two times greater than “side effects,” the second-most frequently identified aspect in this category (Table S9 in Multimedia Appendix 3). In general, aggregate results combining data for all five hashtags were consistent with results for the individual hashtags #CMV and #cytomegalovirus; however, as described here, distinct trends regarding thematic content were observed when quantified by individual hashtags.

### The Impact of the National CMV Awareness Month on Thematic Content of Posts

We next assessed the effect of the National CMV Awareness Month (June 2023) on the thematic content of Twitter/X posts. In our sample of 12,910 (86.6%) posts from May to July 2023, a significant association was observed between the month of publication and the target audience (*χ*^2^_2_=144.3, *P*<.001), with an above-expected increase in the general population in June and a decrease in scientists/health care professionals (adjusted Pearson residuals; see Table S10 in Multimedia Appendix 3). This shift from scientists/health care professionals to the general population from May to June was lost in July (Tables S11-S13 in Multimedia Appendix 3). The population discussed also shifted during the National CMV Awareness Month, with a 110% increase in the “pediatrics” group (n=577, 4.5%, posts in May to n=1213, 9.4%, in June), a 134% increase in “women” (n=280, 2.2%, posts in May to n=656, 5.1%, in June), and a decrease of –32% in “transplant recipients” (n=569, 4.4%, posts in May to n=386, 3%, in June), as shown in Tables S11-S13 in Multimedia Appendix 3. Given the goal of the National CMV Awareness Month to bring awareness about CMV and prevent infection, we next tested for an association between the month of publication and the number of posts that contained these attributes. A significant association was observed between “awareness” and “prevention” and the publication month (*χ*^2^_2_=107.8, *P*<.001), with a significant increase in posts containing awareness messaging compared to that expected and a significant decrease in prevention messaging in June (adjusted Pearson residuals; see Table S10 in Multimedia Appendix 3). To understand which awareness and prevention aspects shifted during the National CMV Awareness Month, we analyzed individual aspects. Within the “awareness and knowledge” thematic category, the absolute number of posts increased across all aspect groups from May to June 2023 (Tables S11-S13 in Multimedia Appendix 3). We then assessed whether the proportional representation of individual aspects also shifted during the National CMV Awareness Month. The proportion of “awareness and knowledge” posts mentioning “burden of disease” increased from 5.7% (59/3773) of posts in May to 9.8% (173/6421) in June (+71%), and posts mentioning “horizontal transmission” increased from 3.8% (39/3773) of posts in May to 10.4% (183/6421) in June (+174%). In contrast, mentions of “universal screening” decreased from 5.6% (58/3773) of posts in May to 3.6% (64/6421) in June (–35%), as shown in Tables S11-S13 in Multimedia Appendix 3. Shifts in the proportional representation of aspects within the “prevention” category also occurred from May to June 2023. The proportion of “prevention” posts with mentions of “vaccine” increased from 24.5% (207/845) of posts in May to 42.9% (368/857) in June (+75%). A proportional decrease in posts that mentioned “antivirals” (–26%; 268/845, 31.7%, posts in May to 202/857, 23.6%, posts in June) and “hygiene measures” (–37%; 50/845, 5.9%, posts in May to 32/857, 3.7%, posts in June) also occurred (Tables S11-S13 in Multimedia Appendix 3). Finally, as expected, the “fundraising” aspect increased within the “general” category of posts during the National CMV Awareness Month (+157%; 37/286, 4.4%, posts in May to 95/329, 11.2%, posts in June), as shown in Tables S11-S13 in Multimedia Appendix 3.

### Sentiment Analyses

**Figure 4:**
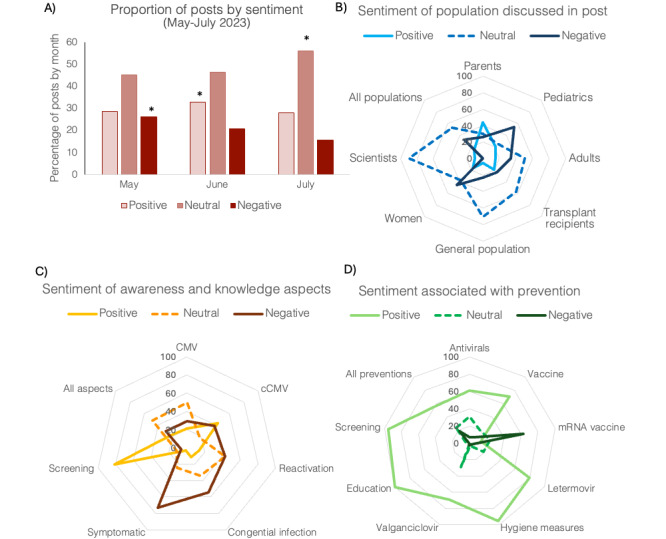
Evaluation of post sentiment from May to July 2023. (a) Proportion of posts scored by overall sentiment per month, (b) by population discussed, (c) by awareness and knowledge, and (d) by type of prevention. Post attributes that occurred infrequently (<10% of the total aspects) were not included. Posts that included more than one attribute are represented in each individual aspect that the posts included. *Significant increase over expected (*P*<.05), chi-square, adjusted Pearson residuals. cCMV: congenital cytomegalovirus; CMV: cytomegalovirus; mRNA: messenger RNA.

Through sentiment analysis, the majority of social media posts were classified as neutral. This was true for most of the broad thematic categories of prespecified aspects (Table S14 in Multimedia Appendix 3 and Figure 4A), though the trend reversed for aspects pertaining to “prevention” for which the number of posts classified as positive (n=1288, 8.6%) was several times greater than the number of posts classified as negative (n=418, 2.8%), as shown in Table S17 in Multimedia Appendix 3. Overall, more social media posts were classified as positive relative to negative (n=3531, 23.7%, vs n=2436, 16.3%, respectively; see Table S14 in Multimedia Appendix 3). In our sample of 12,910 (86.6%) posts from May to July 2023, a significant association was observed between sentiment type and the month of publication (*χ*^2^_4_=163.6, *P*<.001). An examination of all possible combinations of posts revealed posts during the National CMV Awareness Month to be significantly more likely to be positive than expected (adjusted Pearson residuals; see Table S14 in Multimedia Appendix 3 and Figure 4A).

The sentiment associated with independent aspects was also scored. A significant association was observed between sentiment type and the specific target audience, either the general population or scientists/health care professionals (*χ*^2^_2_= 481.2, *P*<.001). Although most posts were classified as neutral (Table S15 in Multimedia Appendix 3), “general population” posts were significantly more likely to be positive or negative in sentiment than expected, while “scientists/health care professionals” posts were significantly more likely to be neutral (adjusted Pearson residuals; see Table S15 in Multimedia Appendix 3).

The sentiment varied depending on the specific “population” discussed,” the specific “awareness and knowledge” aspect, or the type of “prevention.” For example, among the populations discussed, the sentiment of the “scientists” aspect was disproportionally neutral (620/691, 89.7%); in contrast, the sentiment of the “parents” aspect was more likely classified as positive (99/225, 44%), and the most common sentiment for the “pediatrics” aspect was negative (1336/2476, 54%), as shown in Table S16 in Multimedia Appendix 3 and Figure 4B. Examples of aspects included in the “awareness and knowledge” category that diverged from the average sentiment included increased positive association with “screening” (266/327, 81.3%) and increased negative association with “symptomatic” (712/977, 72.9%) and “congenital infection” (437/800, 54.6%), as shown in Table S17 in Multimedia Appendix 3 and Figure 4C. Lastly, “education,” “screening,” and “hygiene measures” aspects included in the “prevention” category were scored as nearly universally positive (education: 23/23, 100%;screening: 22/23, 95.7%; and hygiene measures: 109/114, 95.6%), as shown in Table S18 in Multimedia Appendix 3 and Figure 4D.

### User Engagement

**Figure 5:**
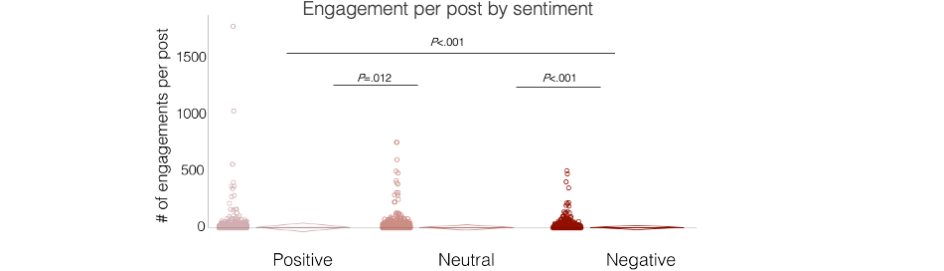
Evaluation of post engagement with respect to sentiment (May-August 2023). A dot plot of the number of engagements per post categorized by sentiment, with the SD displayed to the right. *P values are adjusted with Bonferroni correction, Kruskal-Wallis, Dunn-Bonferroni test. One outlier value (3835, negative) was removed from the graph to improve visualization of data.

In our final analysis, we assessed user engagement with CMV-relevant posts, including “likes,” retweets, and comments. Overall, users engaged with 3863 (27.3%) of 14,136 posts from May to August 2023. Post engagement was slightly higher on average if scored with a positive sentiment (1230/3895, 31.6%) compared to a neutral (1923/6445, 29.8%) or a negative (710/3796, 18.7%) sentiment (Table S19 in Multimedia Appendix 3). To understand potential differences in the magnitude of engagement with respect to sentiment, we compared median engagement per post across sentiment groupings. Engagement was found to be significantly different between sentiments (Kruskal-Wallis; *χ*^2^_2_=194.31, *P*<.001), with a higher rank mean for positive posts compared to neutral and negative posts (Table S19 in Multimedia Appendix 3 and Figure 5). Therefore, posts with a positive sentiment were more likely to engage the audience and to a greater degree, while those with negative sentiment were least likely to do so.

## Discussion

### Principal Findings

The objective of our study was to monitor the volume and thematic content of social media posts on Twitter/X before, during, and after the National CMV Awareness Month in June 2023 to understand the virtual impact of the campaign. We first used a language detection model and a customized BERT tokenizer to extract English language tweets and to remove posts that were not relevant to CMV or CMV disease. Analysis of the remaining 14,900 CMV-relevant posts revealed a peak in posts during the National CMV Awareness Month, a trend observed across all five of the most frequently used CMV-related hashtags, with the highest volume of posts originating from the United States. As expected, these data confirm an active campaign initiative by multiple CMV stakeholders in the United States. We sought to further characterize who is generating information and how effectively their messages are disseminated by identifying the key users and classifying these authors by affiliation. These data point to a potential opportunity to enhance collaboration between advocacy organizations, academic researchers (who were observed to be the most prolific authors), and media outlets (observed to have the largest number of followers) to expand messaging and to target messaging to specific audiences.

Our analyses also examined the thematic content, or aspects, of social media posts, along with the sentiment of each post. A subset of posts (outside of the primary dataset) was annotated by researchers for these attributes, and this dataset was provided to a ChatGPT model, which annotated CMV-relevant posts in substantial agreement with blinded reviewers. Highly mentioned aspects were typically shared between hashtags, though hashtag-specific trends point to specific conversations being siloed to separate channels. For example, “screening” was mentioned primarily under the #stopCMV hashtag, while “transplant recipients” were discussed under the #CMV and #cytomegalovirus hashtags. The National CMV Awareness Month shifted CMV conversations toward the general audience from scientists and health care professionals and were more likely to contain awareness messaging indicating an effort by stakeholders to increase attention with respect to pediatric populations, women, and the burden of disease, most likely reflecting advocacy around cCMV. The sentiment of CMV-relevant social media posts was overall neutral, though it shifted meaningfully toward positive during the campaign month. The overwhelmingly positive sentiment associated with CMV the “prevention” aspects “education,” “screening,” and “hygiene measures” speaks to the enthusiastic advocacy of the CMV community for various interventions and preventive measures. Unexpectedly, prevention messaging decreased significantly during the awareness month even as posts containing prevention aspects were observed to have a positive sentiment, which correlates with higher community engagement.

The methodology used in this study was implemented with multiple checks to ensure accuracy in processing and analysis and is described in sufficient detail to support reproducibility. This allows our methodology to be easily used to evaluate future CMV awareness campaigns to identify long-term trends or shifts in CMV thematic content and sentiment. We envision the possibility of monitoring social media posts as new interventions become available, as is done commonly for sentiment analysis around vaccines against influenza and SARS-CoV-2. Our methods are adaptable and can be expanded to monitor messaging once a vaccine against CMV becomes available or as new legislation is proposed around newborn screening for cCMV. These surveillance and data collection steps provide a foundation for more rigorous interpretation through the lens of health communication and health behavior theory to inform future advocacy and awareness campaigns.

### Limitations

This study has several limitations. We examined posts from a single social media platform (ie, Twitter/X) using Keyhole. Users of Twitter/X are not necessarily representative of the broader US population or all CMV stakeholders; moreover, user demographics likely differ on other platforms, such as Instagram, Reddit, Facebook, and TikTok. Furthermore, although Keyhole is widely used in commercial practice, it is not commonly used in public health studies, in part due to cost. The platform aligns well with real-world social listening practice (eg, real-time tracking), making it suitable for evaluating a public health awareness campaign; however, data were collected, filtered, and summarized by Keyhole rather than accessed as raw posts directly from the Twitter/X API, which may affect completeness and reproducibility.

We were also unable to analyze nearly 9000 non-English tweets, which may differ in terms of author category, aspects, and sentiment relative to the English-language posts analyzed here. Furthermore, the manual classification of aspects and sentiment by researchers is a subjective process, and the list of aspects generated by the authors, while detailed and broad, may not reflect all possible themes discussed in posts. Although moderate in agreement, the interrater reliability in sentiment scores between the four independent raters reflects a limitation in the sentiment analysis. Complete disagreement between the reviewers occurred in 4 of 50 test tweets (positive or negative, excluding neutral), which may reflect the inherently mixed nature of disease-related posts that combine positive messaging around interventions or milestones and negative messaging that describes the underlying need for the intervention. Accurate annotation and classification by AI were dependent on these subjective processes. They may have also been impacted by the constrained length of tweets, which can limit content, explanatory details, and other cues. Lastly, although the pre- and postperiods surrounding June 2023 we examined are limited, we were able to evaluate the immediate impact of the National CMV Awareness Month. For these reasons, the results reported here should be viewed as exploratory and interpreted with this lens. Additional research leveraging the expertise of diverse stakeholders is needed to design and evaluate long-term public health information and future CMV awareness campaigns.

### Strengths

This study also has several notable strengths. To the best of our knowledge, it represents the first systematic infodemiologic evaluation of the National CMV Awareness Month and the first large-scale characterization of conversations concerning CMV on Twitter/X. By analyzing nearly 15,000 CMV-relevant posts, this study leveraged a larger and more comprehensive dataset than would have been feasible to analyze through manually annotation alone. Furthermore, analysis from multiple lenses, such as user characteristics, thematic content, and sentiment, provides a multidimensional view of CMV discussions that has not been unavailable to researchers, advocates, or other relevant stakeholders.

A second strength is the practical demonstration of a multistep analytical pipeline that combines human annotation, model customization, and iterative validation. Furthermore, the application of few-shot prompting with ChatGPT enabled efficient classification of aspects and sentiment at scale. Importantly, human-AI agreement of post sentiment was substantial, increasing confidence in the reliability of the automated post annotations. Finally, the study’s design, which evaluated posts before, during, and after the campaign month, allows for a direct observation of how a nationally recognized campaign shifts online attention, thematic content, and sentiment.

### Conclusion

To the best of our knowledge, this study is the first to examine and report the volume, thematic content, and sentiment of virtual CMV-related conversations on Twitter/X before, during, and after the National CMV Awareness Month. The use of AI permitted detailed evaluation of many thousands of social media posts. The results of our analyses enable us to predict potential collaborations between key users to achieve greater dissemination and impact during future campaigns. In addition, the detailed analyses presented here provide a more complete characterization of the conversations and culture within distinct CMV-related hashtags and highlight the thematic content that can be amplified in future campaigns. The complexity of health communication via social media poses distinct challenges to public health investigators and practitioners when planning and executing information and awareness campaigns. Although this study demonstrates the analytic capabilities of AI, the generative capabilities of the ChatGPT model could also be used to draft campaign messaging to enhance specific themes or emotional undertones.

## Appendix

Multimedia Appendix 1 Expanded methodology.

Multimedia Appendix 2 ChatGPT-4 model master prompt used to annotate aspects and sentiments and identify tweet segments associated with those aspects and sentiments.

Multimedia Appendix 3 Thematic categories, social media post counts, top 20 social media authors, adjusted Pearson residuals and chi-square test results, and engagement with respect to sentiment.

## Abbreviations

AI: artificial intelligence
API: application programming interface
BERT: Bidirectional Encoder Representations from Transformers
cCMV: congenital cytomegalovirus.
CMV: cytomegalovirus
LLM: large language model
NCMVF: National Cytomegalovirus Foundation

## Data Availability

The datasets generated and analyzed during this study are available from the corresponding author upon reasonable request.
