# Supplementary material for: Using Artificial Intelligence Methods to Evaluate the Effect of the National Cytomegalovirus Awareness Month on the Content and Sentiment of Social Media Posts: Infodemiology Study
Source: JMIR Infodemiology. 2026 Jan 22;6:e80922. doi: 10.2196/80922 (PMC12877745; doi:10.2196/80922)
Supplement: Multimedia Appendix 1 [file infodemiology_v6i1e80922_app1.docx]

**Multimedia Appendix 1**

**Expanded BERT Customized Tokenizer Methods**

**Custom BERT Dictionary and Tokenization Procedure**

The BERT tokenizer employs the WordPiece algorithm to segment text into subword units, balancing between word-level and character-level tokenization. During exploratory data analysis, we found that several domain-specific terms and abbreviations were segmented into individual characters or partial tokens (e.g., “CMV” → “C,” “##M,” “##V”; “congenital” → “con,” “##gen,” “##ital”).

Because these terms carry distinct biomedical meaning, character-level fragmentation was undesirable. Therefore, we manually curated a domain-specific dictionary containing frequently used terms relevant to our dataset, including CMV, cCMV, Cytomegalovirus, SARSCoV2, HIV, EBV, HHV, Congenital, and Letermovir. These were added as new tokens to the tokenizer vocabulary so that the terms would be recognized as intact words rather than decomposed subword units.

**Example Tokenization Output**

**Original text:**
“Congenital CMV awareness month. cCMV in results from a pregnant person contracting”

**Default BERT tokenizer:**
“Con”, “##gen”, “##ital”, “c”, “##CM”, “##V”, “awareness”, “month”, “c”, “##CM”, “##V”, “in”, “results”, “from”, “a”, “pregnant”, “person”, “contracting”

**Customized BERT tokenizer:**
“Congenital”, “cCMV”, “awareness”, “month”, “cCMV”, “in”, “results”, “from”, “a”, “pregnant”, “person”, “contracting”

**Impact on Tokenization Accuracy and Model Performance**

To assess the impact of custom tokenization on downstream performance, we fine-tuned two separate models, one using the default BERT tokenizer and one using the customized tokenizer, on the same manually labeled relevance-classification dataset. Both models achieved comparable accuracy (95%), indicating that the custom dictionary did not affect overall classification performance.

Although accuracy differences were minimal, the customized tokenizer produced more interpretable tokenization for biomedical terminology, which may be advantageous for future work and downstream tasks. Because the performance difference was small, additional ablation experiments were not conducted.

To mitigate potential imbalance, the few-shot examples were intentionally constructed to represent a balanced spread across sentiment categories and CMV-related aspect groups (e.g., population, awareness, prevention), reducing the likelihood of systematic bias toward any particular class.
