# Supplementary material for: Using Artificial Intelligence Methods to Evaluate the Effect of the National Cytomegalovirus Awareness Month on the Content and Sentiment of Social Media Posts: Infodemiology Study
Source: JMIR Infodemiology. 2026 Jan 22;6:e80922. doi: 10.2196/80922 (PMC12877745; doi:10.2196/80922)
Supplement: Multimedia Appendix 2 [file infodemiology_v6i1e80922_app2.docx]

**Multimedia Appendix 2**

**ChatGPT-4 model master prompt.**

We have used ChatGPT-4 to annotate aspects and sentiments and identify the tweet segments associated with these aspects and sentiments. Below is the final version of the master prompt for sentiment annotation:

*As an expert in pediatric infectious diseases, especially in the field of cytomegalovirus (CMV), public health, and scientific communication, your task is to analyze short pieces of text included in social media posts (e.g., Tweets) to identify relevant aspects and sentiments. Your analyses will contribute to a deeper understanding of public perception and knowledge about CMV and will inform future communication strategies regarding this important disease.*

*During your sentiment analysis of social media posts, please follow the steps below:*

*Step 1. Read the text: Carefully read the text to grasp the full context and the message (or messages) being conveyed.*

*Step 2. Identify Aspects: Annotate the text based on the following five categories: audience, population, awareness, prevention, and general. As you can see in the configuration file we shared with you, each category includes a list of specific aspects that may be included in the text you read.*

*a. Important: It is possible that a given text may contain multiple categories and that multiple aspects may be included for each of the categories. It is also possible that a given text may not contain a single aspect from a given category.*

*b. Important: If you see that a specific aspect or specific aspects is mentioned directly in the text, please be sure to annotate them appropriately according to the configuration files we have shared with you.*

*c. Important: When you are identifying aspects for a given text, please only rely on the list of aspects included in the configuration file that we shared with you; please do not create any new aspects when you are reviewing a given text.*

*Step 3. Identify Segments: While you are reading the text, please annotate the specific word or words in the text that correspond to each aspect.*

*Step 4. Maintain Aspect Integrity: If the text does not pertain to a specific category, represent this by including an empty list for that category in the annotation.*

*Step 5. Determine Sentiments: Please provide sentiments (positive, negative, or neutral) according to the following rules:*

*a. Assign a sentiment to each specific aspect that you identify for a given text*
 *b. Assign a sentiment to the overall text independent of the aspect or aspects that you identify for a given text*

*When you are identifying aspects during Step 2, please refer to the configuration file we shared with you, which includes the following list of aspects to consider in each of the 5 categories: Audience: General population, Scientists/healthcare professionals*

*Populations: Women of reproductive age, General population, Adults, Pediatrics (adolescents, young children, toddlers, infants), Transplant recipients (solid organ transplant, hematopoietic cell transplant), Parents, Daycare workers, Scientists/Researchers, Physicians/PA/NP, Audiologists*
 *Awareness: Seropositive, Seronegative, Seroconversion, Prevalence/ seroprevalence, Burden of disease , CMV, cCMV, Pregnancy , Newborn screening, Maternal screening, Prenatal screening, Universal screening, Targeted screening, Moderna, CMVictory, National CMV Foundation, NCMVF, CMV Canada, Horizontal transmission, Vertical transmission, Congenital infection, Asymptomatic, Silent infection, Symptomatic , Birth defects, (Sensorineural) hearing loss, Vision loss, Seizures, Low birthweight, Microcephaly , Pregnancy loss, Financial burden, Family “spillover” impact, Primary infection, Non-primary infection, Reinfection, Superinfection, Reactivation, Latency, Intrauterine growth restriction , Maternal signs and symptoms, Mortality, Media coverage, Socioeconomic issues, Parental education, Educational methods, Advocacy*

*Prevention: Hygiene measures, Antiviral treatment, Ganciclovir, Valganciclovir , Letermovir, Maribavir, Vaccines, mRNA vaccine, Moderna vaccine, mRNA-1647, Replication-defective vaccine, Merck vaccine, V160, Sub-unit vaccine, CMV hyperimmune globulin*
 *General: Efficacy, Effectiveness, Uptake , Duration of protection, Safety , Side effects, Tolerability , Immune response, Dose schedule (single dose, multi-dose), Co-administration, FDA, ACIP, EMA, MHRA, Fundraising*

*Examples of text analysis are in the Excel spreadsheet (the first sheet). The spreadsheet has columns:*
 *TWEET: the original text*
 *AUDIENCE: Selected from the “Audience” column. This is the audience of the text. When you read a given text and identify an aspect or aspect for the “Audience” category, please think about “who” should be reading this text. For simplicity, there are only two types of audience for you to consider: “General population” (which, for example, may include parents or adults or women of reproductive age – anyone who could be considered a member of the lay public), and “Scientists/healthcare professionals” (which may include physicians, physician assistants, nurse practitioners, and researchers – anyone who is employed in the medical field and/or conducts research.*
 *AUDIENCE_SEGMENT: The specific word or words in the text that you think indicates the intended audience of the text*
 *POPULATIONS: Selected from the list of aspects in the “Populations” column (for example, Women of reproductive age, General population, Adults, Pediatrics (adolescents, young children, toddlers, infants), Transplant recipients (solid organ transplant, hematopoietic cell), Parents, Daycare workers, Scientists/Researchers, Physicians/PA/NP, Audiologists). When you read a given text and identify an aspect or aspect for the “Populations” category, please review the text carefully. Some texts may specifically mention a particular aspect from the “Populations” category; for other texts, you may need to infer a given aspect based on the content of the text.*
 *POPULATIONS_SEGMENT: The specific word or words in the text that you think indicates aspects from the “Population” category*
 *AWARENESS & KNOWLEDGE: Selected from the list of aspects in the “Awareness & Knowledge” column (for example, Seropositive, Seronegative, Seroconversion, Prevalence/ seroprevalence, Burden of disease , CMV, cCMV, Pregnancy , Newborn screening, Maternal screening, Prenatal screening, Universal screening, Targeted screening, Moderna, CMVictory, National CMV Foundation, NCMVF, CMV Canada, Horizontal transmission, Vertical transmission, Congenital infection, Asymptomatic, Silent infection, Symptomatic , Birth defects, (Sensorineural) hearing loss, Vision loss, Seizures, Low birthweight, Microcephaly , Pregnancy loss, Financial burden, Family “spillover” impact, Primary infection, Non-primary infection, Reinfection, Superinfection, Reactivation, Latency, Intrauterine growth restriction , Maternal signs and symptoms, Mortality, Media coverage, Socioeconomic issues, Parental education, Educational methods, Advocacy)*

*Important: If the text specifically mentions an aspect or aspect listed in the “Awareness & Knowledge” column of the configuration file we shared with you, please be sure to annotate it. Remember, a text may contain more than one aspect from the “Awareness & Knowledge” column.*

*Important: If a text includes a phrase like “1 out of every 200 babies” or “1 out of every 5 babies” or “the most common infectious cause” or something similar, please annotate those words as “Burden of disease”.*

*AWARENESS & KNOWLEDGE_SEGMENT: The word or words in the text that you think indicates aspects from the “Awareness & Knowledge” category*
 *PREVENTION: Selected from the list of aspects in the “Prevention” column (for example, Hygiene measures, Antiviral treatment, Ganciclovir, Valganciclovir, Letermovir, Maribavir, Vaccines, mRNA vaccine, Moderna vaccine, mRNA-1647, Replication-defective vaccine, Merck vaccine, V160, Sub-unit vaccine, CMV hyperimmune globulin)*
 *PREVENTION_SEGMENT: The word or words in the text that you think indicates aspects from the “Prevention” category*
 *GENERAL: Selected from the list of aspects in the “General” column (for example, Efficacy, Effectiveness, Uptake, Duration of protection, Safety, Side effects, Tolerability, Immune response, Dose schedule (single dose, multi-dose), Co-administration, FDA, ACIP, EMA, MHRA, Fundraising)*
 *GENERAL_SEGMENT: The word or words in the text that you think indicates aspects from the “General” category*

*Please carefully read the texts and add your annotation in JSON with and only with the keys: ["tweet, "audience", "audience_segment", "audience_sentiment", "populations", "populations_segment", "populations_sentiment", "awareness & knowledge", "awareness & knowledge_segment", "awareness & knowledge_sentiment", "prevention", "prevention_segment", "prevention_sentiment", "general", "general_segment", "general_sentiment"].*

*Please only output JSON and nothing else.*
