# Supplementary material for: Using Artificial Intelligence Methods to Evaluate the Effect of the National Cytomegalovirus Awareness Month on the Content and Sentiment of Social Media Posts: Infodemiology Study
Source: JMIR Infodemiology. 2026 Jan 22;6:e80922. doi: 10.2196/80922 (PMC12877745; doi:10.2196/80922)
Supplement: Multimedia Appendix 3 [file infodemiology_v6i1e80922_app3.docx]

**Table S1: Five thematic categories and corresponding pre-specified aspects.**

| **Thematic Category** | **Pre-specified Aspects** |
| --- | --- |
| **Target audience** | *General population* (may include parents or adults or women of reproductive age – anyone who could be considered a member of the lay public), and *Scientists/Healthcare professionals* (may include physicians, physician assistants, nurse practitioners, and researchers) |
| **Population discussed** | *Women of reproductive age*, *General population*, *Adults*, *Pediatrics* (adolescents, young children, toddlers, infants), *Transplant recipients* (solid organ transplant, hematopoietic cell), *Parents*, *Daycare workers*, *Scientists/Researchers*, *Physicians/PA/NP*, *Audiologists* |
| **Awareness and knowledge** | *Seropositive*, *Seronegative*, *Seroconversion*, *Prevalence/seroprevalence*, *Burden of disease*, *CMV*, *cCMV*, *Pregnancy*, *Newborn screening*, *Maternal screening*, *Prenatal screening*, *Universal screening*, *Targeted screening*, *Moderna*, *CMVictory*, *National CMV Foundation*, *NCMVF*, *CMV Canada*, *Horizontal transmission*, *Vertical transmission*, *Congenital infection*, *Asymptomatic*, *Silent infection*, *Symptomatic*, *Birth defects*, *(Sensorineural) hearing loss*, *Vision loss*, *Seizures*, *Low birthweight*, *Microcephaly*, *Pregnancy loss*, *Financial burden*, *Family “spillover” impact*, *Primary infection*, *Non-primary infection*, *Reinfection*, *Superinfection*, *Reactivation*, *Latency*, *Intrauterine growth restriction*, *Maternal signs and symptoms*, *Mortality*, *Media coverage*, *Socioeconomic issues*, *Parental education*, *Educational methods*, *Advocacy* |
| **Prevention** | *Hygiene measures*, *Antiviral treatment*, *Ganciclovir*, *Valganciclovir*, *Letermovir*, *Maribavir*, *Vaccines*, *mRNA vaccine*, *Moderna vaccine*, *mRNA-1647*, *Replication-defective vaccine*, *Merck vaccine*, *V160*, *Sub-unit vaccine*, *CMV hyperimmune globulin* |
| **General CMV information** | *Efficacy*, *Effectiveness*, *Uptake*, *Duration of protection*, *Safety*, *Side effects*, *Tolerability*, *Immune response*, *Dose schedule* (single dose, multi-dose), *Co-administration*, *FDA*, *ACIP*, *EMA*, *MHRA*, *Fundraising* |

**Table S2: Social media post count per month by hashtag.**

| **Post/Month** |  |  |  |  | **Percent Change** |
| --- | --- | --- | --- | --- | --- |
|  | **MAY** | **JUNE** | **JULY** | **AUGUST** | **MAY to JUNE** |
| #CMV | 2,370 | 2,970 | 1,900 | 2,434 | 25.3 |
| #CMVawareness | 41 | 174 | 35 | 30 | 324.4 |
| #StopCMV | 61 | 359 | 47 | 62 | 488.5 |
| #cytomegalovirus | 644 | 1321 | 982 | 611 | 105.1 |
| #cCMV | 220 | 356 | 160 | 123 | 61.8 |
| All hashtags | 3,336 | 5,180 | 3,124 | 3,260 | 55.3 |

**Table S3: Number of the top 20 social media authors by category by number of posts or number of followers.**

| **Who is Posting** | **Academia** | **Parents/**  **Public** | **News/**  **Education** | **NGO/**  **Advocacy** | **Physicians/**  **Hospitals** | **Biomedical**  **Industry** | **Government** | **Unsure** |
| --- | --- | --- | --- | --- | --- | --- | --- | --- |
| #CMV | 4 | 3 | 3 | 4 | 4 | 2 | 0 | 3 |
| #CMVAwareness | 6 | 4 | 4 | 3 | 2 | 1 | 1 | 0 |
| #StopCMV | 6 | 4 | 0 | 4 | 4 | 3 | 0 | 0 |
| #cytomegalovirus | 2 | 2 | 5 | 5 | 1 | 1 | 1 | 1 |
| #cCMV | 5 | 4 | 4 | 3 | 2 | 2 | 1 | 0 |
| All Hashtags | 12 | 8 | 11 | 7 | 9 | 4 | 2 | 5 |
|  |  |  |  |  |  |  |  |  |
| **User Influence**  **(Followers)** | **Academia** | **Parents/**  **Public** | **News/**  **Education** | **NGO/**  **Advocacy** | **Physicians/**  **Hospitals** | **Biomedical**  **Industry** | **Government** | **Unsure** |
| #CMV | 1 | 0 | 14 | 0 | 1 | 1 | 3 | 0 |
| #CMVAwareness | 2 | 0 | 10 | 1 | 3 | 1 | 5 | 0 |
| #StopCMV | 5 | 4 | 5 | 2 | 3 | 2 | 1 | 0 |
| #cytomegalovirus | 1 | 0 | 14 | 1 | 1 | 2 | 1 | 2 |
| #cCMV | 1 | 1 | 6 | 1 | 2 | 2 | 7 | 1 |
| All Hashtags | 8 | 5 | 33 | 5 | 9 | 5 | 13 | 3 |

**Table S4: Social media post count by hashtag by target audience of post (all months).**

| ***TARGET AUDIENCE OF POST*** | **Scientists/Healthcare Professionals** | **General Population** |
| --- | --- | --- |
| #CMV | 5,207 | 3,813 |
| #CMVAwareness | 66 | 187 |
| #StopCMV | 75 | 408 |
| #Cytomegalovirus | 1,851 | 1,626 |
| #cCMV | 313 | 415 |
| All Hashtags | 7,575 | 6,449 |

**Table S5: Social media post count by hashtag by population discussed in post (all months).**

| ***POPULATION DISCUSSED IN* *POST*** | **Parents** | **Pediatrics** | **Adults** | **Transplant**  **Recipients** | **General**  **Population** | **Women** | **Scientists**  **Researchers** | **Daycare**  **Workers** | **Physicians**  **PA/NP/etc.** | **Audiologist** |
| --- | --- | --- | --- | --- | --- | --- | --- | --- | --- | --- |
| #CMV | 103 | 1,053 | 450 | 1,217 | 2,184 | 591 | 680 | 20 | 51 | 13 |
| #CMVAwareness | 28 | 104 | 3 | 0 | 4 | 50 | 0 | 0 | 0 | 0 |
| #StopCMV | 24 | 224 | 8 | 1 | 8 | 27 | 2 | 0 | 0 | 1 |
| #Cytomegalovirus | 45 | 555 | 321 | 359 | 524 | 343 | 9 | 1 | 3 | 4 |
| #cCMV | 25 | 520 | 2 | 59 | 7 | 130 | 0 | 0 | 4 | 9 |
| All Hashtags | 225 | 2,456 | 784 | 1,636 | 2,727 | 1,141 | 691 | 21 | 58 | 27 |

**Table S6: Social media post count by hashtag by awareness/knowledge aspect (all months).**

| **AWARENESS AND KNOWLEDGE** | **CMV** | **cCMV** |
| --- | --- | --- |
| #CMV | 7,771 | 555 |
| #CMVAwareness | 166 | 86 |
| #StopCMV | 392 | 64 |
| #Cytomegalovirus | 2,359 | 387 |
| #cCMV | 241 | 506 |
| All Hashtags | 10,929 | 1,598 |

**Table S7: Social media post count by hashtag by awareness/knowledge aspect (other than CMV or cCMV, all months).**

| **AWARENESS AND KNOWLEDGE** | **Reactivation** | **Congenital**  **Infection** | **Symptomatic** | **Hearing loss**  **Sensorineural** | **Burden of disease** | **Horizontal Transmission** | **Prevalence** | **Newborn Screening** | **Universal Screening** | **CMV Canada** |
| --- | --- | --- | --- | --- | --- | --- | --- | --- | --- | --- |
| #CMV | 1,091 | 297 | 621 | 115 | 178 | 46 | 127 | 87 | 63 | 28 |
| #CMVAwareness | 1 | 47 | 10 | 11 | 17 | 1 | 23 | 12 | 9 | 11 |
| #StopCMV | 1 | 9 | 11 | 10 | 4 | 0 | 4 | 21 | 21 | 14 |
| #Cytomegalovirus | 222 | 187 | 292 | 68 | 105 | 205 | 77 | 24 | 12 | 5 |
| #cCMV | 0 | 260 | 43 | 77 | 77 | 46 | 28 | 29 | 43 | 15 |
| All Hashtags | 1,315 | 800 | 977 | 281 | 381 | 298 | 259 | 173 | 148 | 73 |

**Table S8: Social media post count by hashtag by prevention aspect (all months).**

| **PREVENTION ASPECTS** | **Antivirals** | **Vaccine** | **mRNA Vaccine** | **Letermovir** | **Hygiene Measures** | **Valganciclovir** | **Education** | **Screening**  **(General)** | **Screening**  **(Universal)** |
| --- | --- | --- | --- | --- | --- | --- | --- | --- | --- |
| #CMV | 664 | 740 | 313 | 232 | 82 | 140 | 0 | 2 | 6 |
| #CMVAwareness | 0 | 1 | 0 | 0 | 8 | 4 | 1 | 0 | 1 |
| #StopCMV | 0 | 6 | 4 | 0 | 4 | 4 | 0 | 0 | 1 |
| #Cytomegalovirus | 181 | 338 | 136 | 100 | 16 | 57 | 0 | 3 | 3 |
| #cCMV | 4 | 30 | 0 | 0 | 4 | 9 | 0 | 2 | 2 |
| All Hashtags | 849 | 1,115 | 453 | 332 | 114 | 214 | 1 | 7 | 13 |

**Table S9: Social media post count by hashtag by general aspect (all months).**

| **GENERAL ASPECTS** | **Immune**  **Response** | **Side Effects** | **Food and Drug Administration** | **Fundraising** | **Efficacy** | **Safety** | **Socioeconomic**  **Issues** | **Effectiveness** |
| --- | --- | --- | --- | --- | --- | --- | --- | --- |
| #CMV | 324 | 218 | 77 | 112 | 97 | 52 | 3 | 47 |
| #CMVAwareness | 0 | 0 | 0 | 10 | 1 | 0 | 0 | 0 |
| #StopCMV | 0 | 0 | 0 | 56 | 1 | 0 | 0 | 0 |
| #Cytomegalovirus | 209 | 12 | 52 | 13 | 20 | 12 | 0 | 2 |
| #cCMV | 0 | 0 | 0 | 14 | 2 | 4 | 0 | 3 |
| All Hashtags | 533 | 230 | 129 | 205 | 121 | 68 | 3 | 52 |

**Table S10: Adjusted Pearson residuals, Chi-square tests comparing publication month to independent categorial variables**

| **AUDIENCE** | **May** | **June** | **July** |  | **Bonferroni *P*-value** |  | **Adjusted Critical Value** |
| --- | --- | --- | --- | --- | --- | --- | --- |
| General Population | -7.2816878 | 4.86637478 | 1.16180074 |  | 0.008 |  | 2.6 |
| Scientists/Healthcare Professionals | 6.71052221 | -4.484663 | -1.0706707 |  |  |  |  |
|  |  |  |  |  |  |  |  |
| **AWARENESS/PREVENTION** | **May** | **June** | **July** |  | **Bonferroni *P*-value** |  | **Adjusted Critical Value** |
| Awareness | -1.3129185 | 3.14160872 | -2.5927308 |  | 0.008 |  | 2.6 |
| Prevention | 2.90329907 | -6.9471409 | 5.73338944 |  |  |  |  |

**Table S11: Social media post count by category in May of 2023 (all hashtags).**

| **MAY** | | | | | | | | | | | | | | |  |  |
| --- | --- | --- | --- | --- | --- | --- | --- | --- | --- | --- | --- | --- | --- | --- | --- | --- |
| **AUDIENCE** | | |  | **POPULATIONS** | |  | **AWARENESS & KNOWLEDGE** | |  | **PREVENTATION** | |  | **GENERAL** | | |  |
| General population | | 1,127 |  | Parents | 26 |  | cCMV | 457 |  | vaccine | 207 |  | immune response | 105 | | |
| Scientists/healthcare professionals | | 1,920 |  | Pediatrics | 577 |  | congenital | 218 |  | antiviral | 268 |  | side effects | 53 | | |
|  |  |  |  | Women | 280 |  | symptomatic | 249 |  | mRNA vaccine | 98 |  | FDA | 70 | | |
|  |  |  |  | Adults | 329 |  | Horizontal | 39 |  | Letermovir | 145 |  | Fundraising | 37 | | |
|  |  |  |  | Audiologist | 4 |  | CMV | 2,373 |  | Hygiene | 50 |  | Efficacy | 10 | | |
|  |  |  |  | Transplant | 569 |  | Universal screening | 58 |  | Valganciclovir | 67 |  | Effectiveness | 1 | | |
|  |  |  |  | General | 264 |  | hearing | 85 |  | Education | 0 |  | safety | 10 | | |
|  |  |  |  | Daycare | 2 |  | Newborn screening | 42 |  | Universal screening | 7 |  | Socioeconomic  Issues | 0 | | |
|  |  |  |  | Physician | 6 |  | reactivation | 193 |  | Newborn screening | 3 |  |  |  | | |
|  |  |  |  | Scientist | 6 |  | burden | 59 |  |  |  |  |  |  | | |
|  |  |  |  |  |  |  | prevalence | 66 |  |  |  |  |  |  | | |
|  |  |  |  |  |  |  | CMV Canada | 22 |  |  |  |  |  |  | | |

**Table S12: Social media post count by category in June of 2023 (all hashtags).**

| **JUNE** | | | | | | | | | | | | | | |  |
| --- | --- | --- | --- | --- | --- | --- | --- | --- | --- | --- | --- | --- | --- | --- | --- |
| **AUDIENCE** | | |  | **POPULATIONS** | |  | **AWARENESS & KNOWLEDGE** | |  | **PREVENTATION** | |  | **GENERAL** | | |
| General population | | 2,456 |  | Parents | 104 |  | cCMV | 864 |  | vaccine | 368 |  | immune response | 109 | |
| Scientists/healthcare professionals | | 2,392 |  | Pediatrics | 1,213 |  | congenital | 307 |  | antiviral | 202 |  | side effects | 25 | |
|  |  |  |  | Women | 656 |  | symptomatic | 310 |  | mRNA vaccine | 83 |  | FDA | 53 | |
|  |  |  |  | Adults | 294 |  | Horizontal | 183 |  | Letermovir | 102 |  | Fundraising | 95 | |
|  |  |  |  | Audiologist | 13 |  | CMV | 3,957 |  | Hygiene | 32 |  | Efficacy | 30 | |
|  |  |  |  | Transplant | 386 |  | Universal screening | 64 |  | Valganciclovir | 62 |  | Effectiveness | 2 | |
|  |  |  |  | General | 344 |  | hearing | 136 |  | Education | 0 |  | safety | 10 | |
|  |  |  |  | Daycare | 13 |  | Newborn screening | 70 |  | Universal screening | 4 |  | Socioeconomic  Issues | 5 | |
|  |  |  |  | Physician | 5 |  | reactivation | 357 |  | Newborn screening | 4 |  |  |  | |
|  |  |  |  | Scientist | 10 |  | burden | 173 |  |  |  |  |  |  | |
|  |  |  |  |  |  |  | prevalence | 124 |  |  |  |  |  |  | |
|  |  |  |  |  |  |  | CMV Canada | 36 |  |  |  |  |  |  | |

**Table S13: Social media post count by category in July of 2023 (all hashtags).**

| **JULY** | | | | | | | | | | | | | | |  |
| --- | --- | --- | --- | --- | --- | --- | --- | --- | --- | --- | --- | --- | --- | --- | --- |
| **AUDIENCE** | | |  | **POPULATIONS** | |  | **AWARENESS & KNOWLEDGE** | |  | **PREVENTATION** | |  | **GENERAL** | | |
| General population | | 1,398 |  | Parents | 67 |  | cCMV | 207 |  | vaccine | 423 |  | immune response | 307 | |
| Scientists/healthcare professionals | | 1,553 |  | Pediatrics | 479 |  | congenital | 236 |  | antiviral | 228 |  | side effects | 127 | |
|  |  |  |  | Women | 154 |  | symptomatic | 285 |  | mRNA vaccine | 256 |  | FDA | 3 | |
|  |  |  |  | Adults | 124 |  | Horizontal | 26 |  | Letermovir | 46 |  | Fundraising | 34 | |
|  |  |  |  | Audiologist | 5 |  | CMV | 2,495 |  | Hygiene | 20 |  | Efficacy | 33 | |
|  |  |  |  | Transplant | 462 |  | Universal screening | 20 |  | Valganciclovir | 43 |  | Effectiveness | 29 | |
|  |  |  |  | General | 1,011 |  | hearing | 41 |  | Education | 1 |  | safety | 24 | |
|  |  |  |  | Daycare | 0 |  | Newborn screening | 32 |  | Universal screening | 1 |  | Socioeconomic  Issues | 0 | |
|  |  |  |  | Physician | 25 |  | reactivation | 653 |  | Newborn Screening | 0 |  |  |  | |
|  |  |  |  | Scientist | 154 |  | burden | 102 |  |  |  |  |  |  | |
|  |  |  |  |  |  |  | prevalence | 43 |  |  |  |  |  |  | |
|  |  |  |  |  |  |  | CMV Canada | 10 |  |  |  |  |  |  | |

**Table S14: Social media post count by month and overall sentiment (all hashtags), with Adjusted Pearson residuals.**

| **Post Count (Overall sentiment)** | **May** | **June** | **July** | **Sum** |  |
| --- | --- | --- | --- | --- | --- |
| Positive | 951 | 1,705 | 875 | 3,531 |  |
| Neutral | 1,514 | 2,402 | 1,757 | 5,673 |  |
| Negative | 871 | 1,073 | 492 | 2,436 |  |
|  |  |  |  |  |  |
| **Adjusted Pearson residuals** | **May** | **June** | **July** | **Bonferroni *P*-value** | **Adjusted Critical Value** |
| Positive | -1.9168271 | 3.37142334 | -2.3605294 | 0.0056 | 2.7 |
| Neutral | -2.774413 | -2.4396841 | 6.00854886 |  |  |
| Negative | 6.54165649 | -0.3359704 | -6.3272537 |  |  |

**Table S15: Social media post count by target audience and sentiment (all hashtags), with Adjusted Pearson residuals.**

| **Post Count (Audience Sentiment)** | **General Population** | **Scientists/Healthcare Professionals** |  |  | |
| --- | --- | --- | --- | --- | --- |
| Positive | 1,462 | 1,106 |  |  | |
| Neutral | 3,691 | 5,390 |  |  | |
| Negative | 863 | 398 |  |  | |
| **Adjusted Pearson residuals** | **General Population** | **Scientists/Healthcare Professionals** | **Bonferroni *P*-value** | | **Adjusted Critical Value** |
| Positive | 7.67135775 | -7.1662262 | 0.0083 | 2.6 | |
| Neutral | -8.3135382 | 7.76612132 |  |  | |
| Negative | 11.362344 | -10.614174 |  |  | |

**Table S16: Social media post count by population discussed and sentiment (all hashtags).**

| **Post Count (Population Sentiment)** | **Parents** | **Pediatrics** | **Adults** | **Transplant** | **General** | **Women** | **Scientist** | **All aspects** |
| --- | --- | --- | --- | --- | --- | --- | --- | --- |
| Positive | 99 | 519 | 117 | 309 | 153 | 192 | 70 | 1,262 |
| Neutral | 67 | 621 | 400 | 933 | 1,935 | 432 | 620 | 4,538 |
| Negative | 59 | 1,336 | 267 | 394 | 639 | 517 | 1 | 2,767 |
| Sum | 225 | 2,476 | 784 | 1,636 | 2,727 | 1,141 | 691 | 8,567 |

**Table S17: Social media post count by awareness/knowledge aspect and sentiment (all hashtags).**

| **Post Count (Awareness Sentiment)** | **CMV** | **cCMV** | **Reactivation** | **Congenital** | **Symptomatic** | **Screening** | **All aspects** |
| --- | --- | --- | --- | --- | --- | --- | --- |
| Positive | 2,306 | 692 | 135 | 90 | 28 | 266 | 2,894 |
| Neutral | 5,421 | 291 | 433 | 273 | 237 | 39 | 6,344 |
| Negative | 3,165 | 615 | 433 | 437 | 712 | 22 | 3,807 |
| Sum | 10,892 | 1,598 | 1,001 | 800 | 977 | 327 | 13,045 |

**Table S18: Social media post count by prevention aspect and sentiment (all hashtags).**

| **Post Count (Prevention Sentiment)** | **Antivirals** | **Vaccines** | **mRNA vaccine** | **Letermovir** | **Hygiene measures** | **Valganciclovir** | **Education** | **Screening** | **All aspects** |
| --- | --- | --- | --- | --- | --- | --- | --- | --- | --- |
| Positive | 519 | 493 | 61 | 265 | 109 | 147 | 23 | 22 | 1,288 |
| Neutral | 267 | 133 | 108 | 62 | 3 | 62 | 0 | 1 | 531 |
| Negative | 63 | 67 | 284 | 5 | 2 | 5 | 0 | 0 | 418 |
| Sum | 849 | 693 | 453 | 332 | 114 | 214 | 23 | 23 | 2,237 |

**Table S19: Engagement with respect to Sentiment (all hashtags).**

| **Ranks** | **n** | **Median** | **Mean Rank** |  |  |
| --- | --- | --- | --- | --- | --- |
| Negative | 3796 | 0 | 6462.34 |  |  |
| Neutral | 6445 | 0 | 7220.37 |  |  |
| Positive | 3895 | 0 | 7407.95 |  |  |
| **Dunn-Bonferroni-Tests** | **Test Statistic** | **Std. Error** | **Std. Test Statistic** | ***P*-value** | **Adjusted *P*-value^a^** |
| Negative - Neutral | -758.03 | 65.51 | -11.57 | <0.001 | <0.001 |
| Negative - Positive | -945.61 | 73.03 | -12.95 | <0.001 | <0.001 |
| Neutral - Positive | -187.58 | 64.99 | -2.89 | 0.004 | 0.012 |
| ***Note: a****. Values adjusted with Bonferroni correction.* | | | |  |  |
